# Solitons in a modified discrete nonlinear Schrödinger equation

**DOI:** 10.1038/s41598-018-20490-2

**Published:** 2018-02-01

**Authors:** Mario I. Molina

**Affiliations:** Departamento de Física and MSI-Nucleus on Advanced Optics Facultad de Ciencias, Universidad de Chile, Casilla 653, Santiago, Chile

## Abstract

We study the bulk and surface nonlinear modes of a modified one-dimensional discrete nonlinear Schrödinger (mDNLS) equation. A linear and a modulational stability analysis of the lowest-order modes is carried out. While for the fundamental bulk mode there is no power threshold, the fundamental surface mode needs a minimum power level to exist. Examination of the time evolution of discrete solitons in the limit of strongly localized modes, suggests ways to manage the Peierls-Nabarro barrier, facilitating in this way a degree of soliton steering. The long-time propagation of an initially localized excitation shows that, at long evolution times, nonlinear effects become negligible and as a result, the propagation becomes ballistic. The qualitative similarity of the results for the mDNLS to the ones obtained for the standard DNLS, suggests that this kind of discrete soliton is an robust entity capable of transporting an excitation across a generic discrete medium that models several systems of interest.

## Introduction

In the semiclassical approach to the coupled electron-phonon problem, the electronic degrees of freedom are coupled to the vibrational ones, where the latter are pictured as classical oscillators. A further approximation assumes that these oscillators are enslaved to the electron, thus reducing the number of equations which now contain only electronic coordinates. When the oscillators are pictured as Einstein or, optical oscillators, one arrives to an effective electronic equation known as the Discrete Nonlinear Schödinger(DNLS) equation^1–12^:

1 $$i\frac{d{C}_{n}}{dt}+V({C}_{n+1}+{C}_{n-1})+\chi |{C}_{n}{|}^{2}{C}_{n}=0.$$

Here, *C*_*n*_ is the electronic probability amplitude at site *n*, *V* is the coupling between nearest neighbor sites, and *χ* is the nonlinearity parameter, proportional to the square of the electron-phonon coupling. Identical equation appears in other systems such as coupled waveguide arrays in optics^8,10,11^, BECs in coupled magneto-optical traps^13,14^ and biomolecules^15–19^, to name a few. On the other hand, when the oscillators are taken as of the Debye, or acoustic type, one arrives to the less-known Modified Discrete Nonlinear (mDNLS) equation:

2 $$i\frac{d{C}_{n}}{dt}+V({C}_{n+1}+{C}_{n-1})+\chi (|{C}_{n+1}{|}^{2}+|{C}_{n-1}{|}^{2}+\mathrm{2|}{C}_{n}{|}^{2}){C}_{n}=0.$$

Hereafter, we will dispense with the physical origin of the mDNLS and proceed to focus on its selftrapping and transport properties, and show that its soliton phenomenology is similar to the one found previously in DNLS. This is very important since it supports the idea that a discrete soliton is a robust excitation of the system, regardless of the precise nature of the underlying vibrational degrees of freedom. In this sense, the DNLS and mDNLS can be regarded as complementary equations.

Equation (2) has two conserved quantities: The power *P* = ∑_*n*_|*C*_*n*_|^2^, and the Hamiltonian

3 $$\begin{array}{rcl}H & = & V\sum _{n}({C}_{n}{C}_{n+1}^{\ast }+{C}_{n}^{\ast }{C}_{n+1})\\  &  & +\chi \sum _{n}(|{C}_{n}{|}^{2}|{C}_{n+1}{|}^{2}+|{C}_{n}{|}^{4}).\end{array}$$

In addition, our system obeys the staggered-unstaggered symmetry: Equation (1) is invariant under the transformation $\chi \to -\chi ,{C}_{n}\to {(-\mathrm{1)}}^{n}{C}_{n}^{\ast }$, where the * denotes the complex conjugate. Our system is a Hamiltonian one since from Eq. (3) one obtains Eq. (1) by using the canonical equations *dq*_*n*_/*dt* = ∂*H*/∂*p*_*n*_, *dp*_*n*_/*dt* = −∂*H*/∂*q*_*n*_ with canonically conjugate variables *q*_*n*_ = *C*_*n*_ and ${p}_{n}=i{C}_{n}^{\ast }$. The mDNLS was first found in earlier studies of polaron formation, from the coupled electron-phonon equations in the adiabatic limit^20,21^. For the case of a large polaron whose size is much larger than the lattice spacing, a continuum approximation is possible and both Eqs (1) and (2), converge to the continuous Nonlinear Schrödinger equation (NLS), which has a well-known soliton solution. The mDNLS has been used to study certain recurrences that occur in the coupled electron-phonon problem^21,22^. The selftrapping properties of the mDNLS were also examined in ref.^22^. The DNLS and mDNLS equations are complementary and are useful to describe the dynamics of excitations.

In this work we will focus on the different families of nonlinear bulk and surface modes and their stability properties, transport exponents and on the propagation of mDNLS solitons, looking for possible means of propagation control.

## Nonlinear modes

The nonlinear modes are found by setting *C*_*n*_(*t*) = *ϕ*_*n*_ exp *iλt*, which leads to the nonlinear eigenvalue equation

4 $$-\lambda {\varphi }_{n}+V({\varphi }_{n+1}+{\varphi }_{n-1})+\chi (|{\varphi }_{n+1}{|}^{2}+|{\phi }_{n-1}{|}^{2}+\mathrm{2|}{\varphi }_{n}{|}^{2}){\varphi }_{n}=0.$$

**Figure 1:**
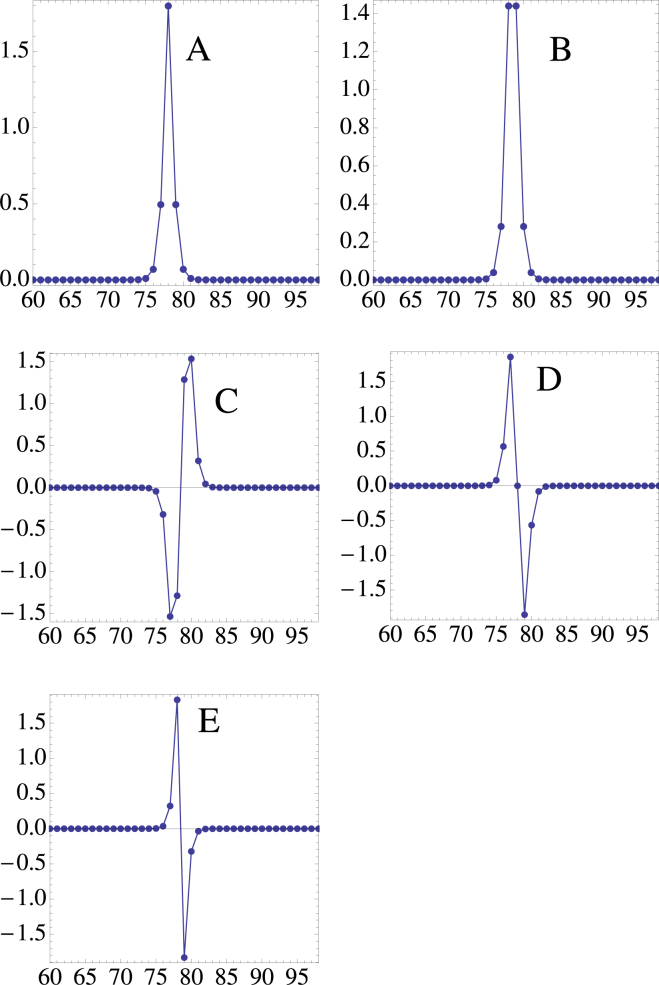
Some lowest-order nonlinear bulk modes (*V* = 1, *λ* = 9.3). The modes shown here have different power content.

**Figure 2:**
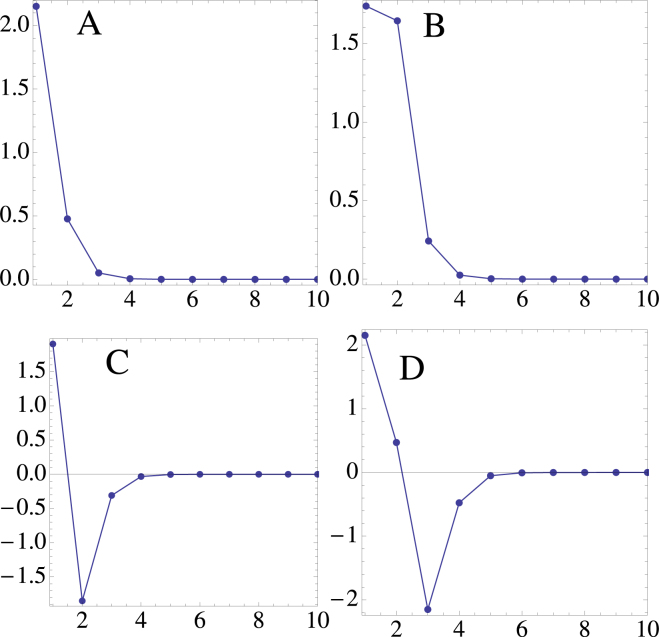
Some lowest-order nonlinear surface modes (*V* = 1, *λ* = 9.3). The modes shown here have different power content.

For a given *λ*, the system of equations (4) is solved numerically by means of a multidimensional Newton-raphson scheme, using as a seed the form obtained from the decoupled limit, also known as the anticontinuous limit. The boundary conditions are open, with lattice sites ranging from *n* = 1 up to *n* = *N*. Hereafter, we will set *χ* > 0; the modes in the case of negative nonlinearity can be obtained through the staggered-unstaggered transformation mentioned above. Figures 1 and 2 show examples of the lowest-order nonlinear localized modes, for the bulk (away from *n* = 1 or *n* = *N*) and the surface (near *n* = 1 or *n* = *N*) respectively. For the bulk case, we see an “odd” mode (A), and “even” mode (B) and three “twisted” modes (C, D and E). For the surface case we see a truncated “odd” mode (A), a “flat top” mode (B) and two “twisted” modes (C and D). It should be mentioned here that other, non-rigurous ‘edge” modes are possible, with maximum lying at *n* = 1, 2, 3 etc. In fact it is possible to transition from a pure edge mode (*n* = 1) to a bulk mode ($n\gg 1$), simply by using the appropriate initial starting point in the Newton-Raphson computation. To compute the linear stability of the edge modes we introduce a weak perturbation as *C*_*n*_(*t*) = (*ϕ*_*n*_ + *δ*_*n*_(*t*)) exp (*iλt*), and obtain a linear evolution equation for *δ*_*n*_(*t*), where $|{\delta }_{n}(t)|\ll |{\varphi }_{n}|$. After decomposing *δ*_*n*_(*t*) = *x*_*n*_(*t*) + *iy*_*n*_(*t*), and inserting into Eq. (1), one arrives at a set of coupled real equations:

5 $$\begin{array}{cc}\frac{d}{dt}\overrightarrow{x}+{\bf{A}}\,\overrightarrow{y}=\mathrm{0,} & \frac{d}{dt}\overrightarrow{y}+{\bf{B}}\,\overrightarrow{x}=0\end{array}$$

where x = (*x*_1_, *x*_2_, ... *x*_*N*_), y = (*y*_1_, *y*_2_, ... *y*_*N*_) and **A** and **B** are matrices defined by

6 $$\begin{array}{rcl}{{\bf{A}}}_{nm} & = & (-\lambda +\chi |{\varphi }_{n+1}{|}^{2}+\chi |{\varphi }_{n-1}{|}^{2}+4\chi |{\varphi }_{n}{|}^{2}-2\chi {\varphi }_{n}^{2}){\delta }_{nm}\\  &  & +(V+\chi {\varphi }_{n}({\varphi }_{n+1}^{\ast }-{\varphi }_{n+1})){\delta }_{n,m-1}\\  &  & +(V+\chi {\varphi }_{n}({\varphi }_{n-1}^{\ast }-{\varphi }_{n-1})){\delta }_{n,m+1}\end{array}$$

and

7 $$\begin{array}{rcl}{{\bf{B}}}_{nm} & = & (\lambda -\chi |{\varphi }_{n+1}{|}^{2}-\chi |{\varphi }_{n-1}{|}^{2}-4\chi |{\varphi }_{n}{|}^{2}-2\chi {\varphi }_{n}^{2}){\delta }_{nm}\\  &  & -(V+\chi {\varphi }_{n}({\varphi }_{n+1}^{\ast }+{\varphi }_{n+1})){\delta }_{n,m-1}+\\  &  & -(V+\chi {\varphi }_{n}({\varphi }_{n-1}^{\ast }+{\varphi }_{n-1})){\delta }_{n,m+1}\end{array}$$

From Eq. (5) one obtains

8 $$\begin{array}{cc}\frac{{d}^{2}}{d{t}^{2}}\overrightarrow{x}-{\bf{A}}{\bf{B}}\,\overrightarrow{x}=\mathrm{0,} & \frac{{d}^{2}}{d{t}^{2}}\overrightarrow{y}-{\bf{B}}{\bf{A}}\,\overrightarrow{y}=0\end{array}$$

Thus, the linear stability of the nonlinear modes is determined by the eigenvalue spectra of the matrices **AB** and **BA**. A convenient parameter to quantify the stability of a mode is the instability gain G, defined as

9 $$G=\,{\rm{Max}}\,{\rm{of}}{\{\tfrac{1}{2}(Re[g]+\sqrt{Re{[g]}^{2}+Im{[g]}^{2}})\}}^{\mathrm{1/2}}$$

over all *g* values, where *g* is one of the eigenvalues of **A B** (or **B A**). When *G* is zero, the mode is stable; otherwise it is unstable. This parameter is nothing else but the largest growth rate of the mode and is given by the imaginary part of the square root of the complex eigenvalue of **A B** (or **B A**).

**Figure 3:**
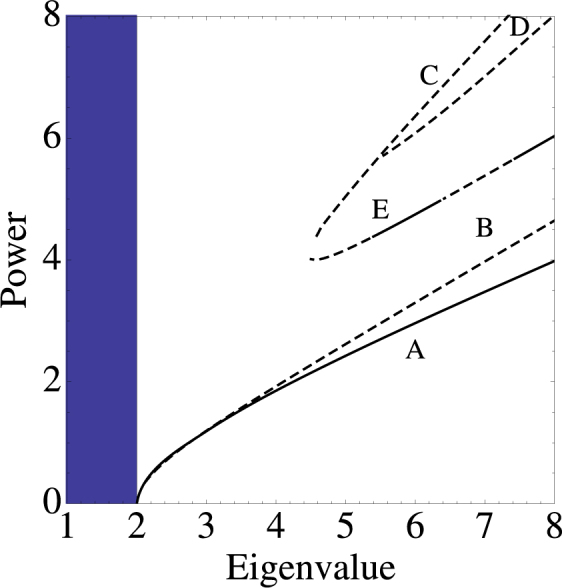
Power content versus eigenvalue for the nonlinear modes of Fig. 1. Continuous (dashed) curves denote stable (unstable) modes (*V* = 1, *χ* = 1).

**Figure 4:**
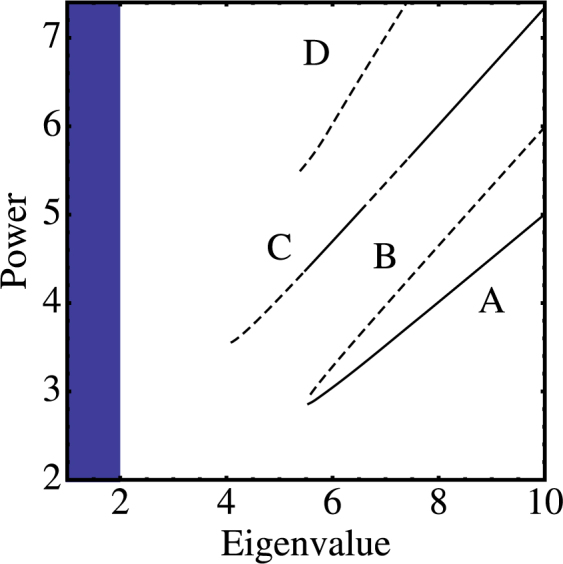
Power content versus eigenvalue for the nonlinear modes of Fig. 2. Continuous (dashed) curves denote stable (unstable) modes. (*V* = 1, *χ* = 1)

Figures 3 and 4 show the power vs eigenvalue curves for some lowest-order modes, along with their stability. We note that, while for the bulk modes, there are at least two modes with no threshold power, for the surface modes they all require a minimum power threshold (nonlinearity) to exist. The only stable lowest-order bulk mode is the odd one, which is stable all the way down to the linear band. For the surface modes we observe that they all require a minimum power threshold to exist.

## Soliton propagation

**Figure 5:**
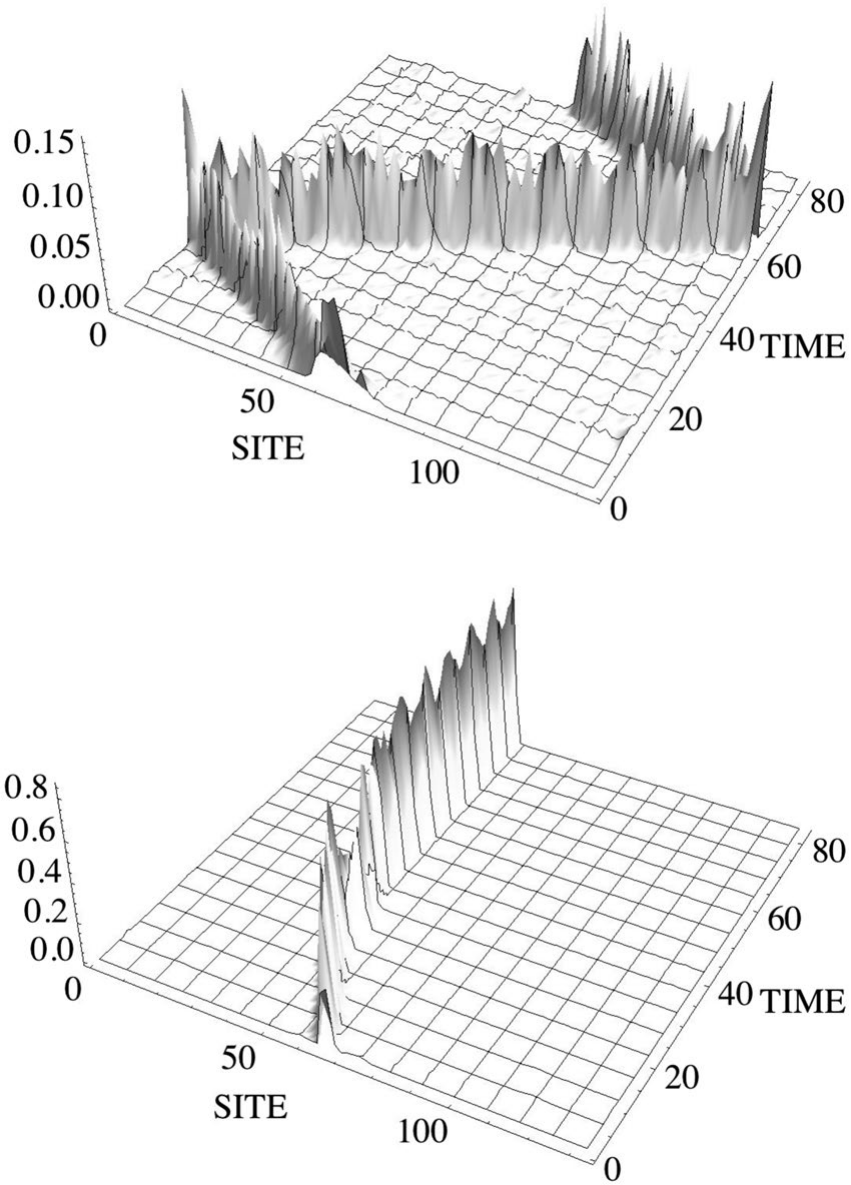
(**a**,**b**) Examples of propagation of a discrete mDNLS soliton for large and small values of the initial momentum. Top: *k* = 0.4, *A* = 0.5. Bottom: *k* = 0.2, *A* = 0.8. For both cases *V* = 1, *χ* = 1.

We consider here the propagation of an approximate soliton solution using Eq. (1). As an initial condition we will use the form $u(0)=A\,{\rm sech} [(A/\sqrt{2})(n-{n}_{c})]\exp [-ik(n-{n}_{c})]$ which is a discretization of the exact continuous NLS one-soliton solution. Parameter *k* represents the initial momentum of the pulse and *n*_*c*_ is the position of the soliton center. This ansatz is reasonable for wide solitons where the discrete character of the lattice is of no consequence. Figure 5 shows an example of discrete soliton propagation for two different values of momentum. In the case of large kick, the soliton propagates across the lattice and bounces elastically from the ends of the chain. For low values of *k*, the soliton propagates some short distance and gets selftrapped eventually around some lattice site. It should be noted that similar results are obtained for more generic spatial profiles that are localized in space and endowed with an initial kick. One example of this is using the profile corresponding to the fundamental stationary mode. In all cases we have the generic behavior that mobility is enhanced for relatively wide profiles, and/or high values of the momentum *k*.

## Propagation control

One of the major problems for achieving controllable steering of discrete solitons is the existence of an effective periodic potential, known as the Peierls-Nabarro (PN) potential, that appears as a result of lattice discreteness. While in the continuous case, the presence of translational invariance favors soliton propagation, in a discrete system a minimum impulse is needed to effect soliton motion. The magnitude of the PN potential can be roughly estimated as *A*^4^, where *A* is the soliton amplitude^23^. We can shed some light into this problem by the use of strongly localized modes (SLMs)^24–26^. We consider a stationary odd SLM in the form

10 $${\varphi }_{n}=\{0,0,\ldots ,\varepsilon \,\exp (-ik),1,\epsilon \,\exp (ik\mathrm{),0,}\,\cdots ,0\}{\varphi }_{o}$$

where $\varepsilon \ll 1$. This mode has an associated Hamiltonian given by

11 $$\begin{array}{rcl}{H}_{odd} & = & -2V{\varphi }_{0}^{2}(\epsilon +{\epsilon }^{\ast })\,\cos (k)-\chi {\varphi }_{o}^{4}\mathrm{(1}+\mathrm{2|}\epsilon {|}^{2}+\mathrm{2|}\epsilon {|}^{4})\\  & \approx  & -\chi {\varphi }_{o}^{4}.\end{array}$$

On the other hand, an even SLM has the form

12 $${\varphi }_{n}=\{0,0,\ldots \,,\epsilon \,\exp (-ik),1,\exp (ik),\epsilon \,\exp \mathrm{(2}ik),\,\mathrm{0,}\,\ldots \,,0\}{\tilde{\varphi }}_{o}$$

and an associated Hamiltonian

13 $${H}_{even}\approx -4V\,\cos (k){{\tilde{\varphi }}_{0}}^{2}-3\chi {{\tilde{\varphi }}_{0}}^{4}$$

On the other hand, the power content of these localized modes is given by

14 $${P}_{odd}\approx {\varphi }_{0}^{2}+O({\varepsilon }^{2})$$

and

15 $${P}_{even}\approx 2{{\tilde{\varphi }}_{0}}^{2}+O({\varepsilon }^{2})$$

Now we assume that the odd and even SLM are different states of the *same* soliton. This implies that both SLMs possess the same norm (power). Therefore, ${\varphi }_{0}^{2}\approx 2{\tilde{\varphi }}_{o}^{2}$. The even Hamiltonian becomes

16 $${H}_{even}\approx -2V\,\cos (k){\varphi }_{0}^{2}-\mathrm{(3/4)}\chi {\varphi }_{0}^{4}.$$

The dynamical barrier can now be defined as the difference Δ = *H*_*odd*_ − *H*_*even*_, that is,

17 $${\rm{\Delta }}\approx 2V{\varphi }_{0}^{2}\,\cos (k)-\mathrm{(1/4)}\chi {\varphi }_{0}^{4}.$$

We see that, to a first approximation, the barrier could be tuned by an appropriate choice of the amplitude, momentum and nonlinearity parameter. For the ideal case Δ = 0 the discrete soliton would propagate unimpeded across the lattice. If our objective is not to effect a free propagation, but to deliver the soliton at a given location (where it will remain due to selftrapping), like in a multiport switching, one could in principle, resort to an engineering of the couplings^27,28^ to bring the soliton from a given position to any desired site.

## Modulational Stability

**Figure 6:**
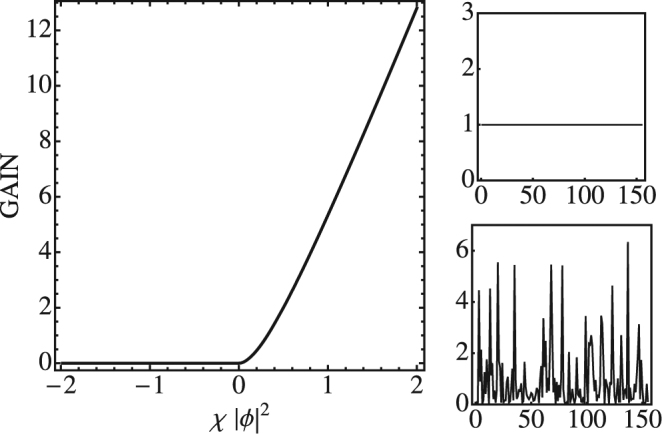
Left: Instability gain versus nonlinearity strength. Right: Snapshot at *Vt* = 133 of initially uniform spatial profile *ϕ* = 1. Top: *χ* < 0. Bottom: *χ* > 0. (*N* = 155, *V* = 1, periodic boundary conditions).

When dealing with the dynamical evolution of the mDLNS equation, it is natural to ask under which circumstance the system will create discrete solitons instead of radiation. A clue about this comes from examining the linear stability of an initially uniform nonlinear profile. When this profile becomes unstable, the profile will tend to fragment and the largest fragments could serve as seeds for discrete solitons. Usually this depends on the strength of nonlinearity. Let us consider a solution of the form *C*_*n*_(*t*) = *ϕ* exp(*iλt*). After inserting this solution into Eq. (1), we conclude *λ* = 2*V* + 4*χϕ*^2^ and therefore *C*_*n*_(*t*) = *ϕ* exp[*i*(2*V* + 4*ϕ*^2^)*t*]. We insert this solution into Eqs (6) and (7) and obtain

18 $$\begin{array}{l}{{\boldsymbol{A}}}_{nm}=-2V{\delta }_{nm}+V({\delta }_{n,m}+{\delta }_{n,m-1})\\ {{\boldsymbol{B}}}_{nm}=\mathrm{(2}V-4\chi {\varphi }^{2}){\delta }_{nm}-(V+2\chi {\varphi }^{2})({\delta }_{n,m+1}+{\delta }_{n,m-1})\end{array}$$

and proceed with an analysis of the gain parameter. This procedure gives the same information as a semi-analytical method^29^. Results are shown in Fig. 6 which shows the instability gain versus the nonlinearity strength. For positive nonlinearity parameter the gain is positive signaling instability of the uniform profile and thus, adequate conditions for the creation of discrete solitons. For negative nonlinearity strength, the gain is identically zero. This results are in agreement with those obtained for the DNLS in the limit of uniform initial profile^29^.

However, since in our case the nonlinearity parameter is proportional to the square of the electron-phonon interaction, it is always positive and we can conclude that the system is modulationally unstable and thus, prone to generating discrete solitons.

## Transport

Finally, let us look at the transport properties of the mDNLS system. The typical thing to do is to examine the mean square displacement of an initially localized initial condition, at long evolution times

19 $${\sigma }^{2}=\frac{\sum _{n}{n}^{2}|{C}_{n}(t{)|}^{2}}{\sum _{n}|{C}_{n}(t{)|}^{2}}.$$

where, *C*_*n*_(0) = *δ*_*n*,0_. At long times, the asymptotic behavior is of the form ${\sigma }^{2} \sim {t}^{\alpha }$, where *α* is the propagation exponent. When *α* = 2 we have ballistic motion, for *α* = 1 we have diffusive motion, for 1 < *α* < 2 we have super-diffusive motion, and for 0 < *α* < 1 we have sub-diffusive motion.

**Figure 7:**
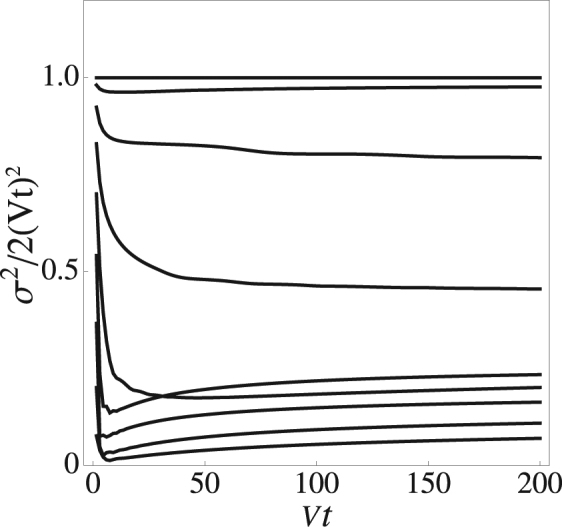
Mean square displacement versus time for several nonlinearity parameter values. From top to bottom: *χ* = 0, 0.5, 1, 1.5, 2, 2.5, 3, 3.5, 4.

Figure 7 shows the evolution of the mean square displacement in time (normalized to the case of a linear chain), for several values of the nonlinearity parameter. We have used a chain with *N* = 900 sites which is sufficiently long to avoid reflection from the boundaries for *Vt* = 200, of a ballistic pulse (the fastest propagation). As we can see, after a transient, all the curves converge to a nearly horizontal line denoting an asymptotic exponent close to 2, as Table 1 shows. This can be explained noting that as time evolves, the profile expands and brings the nonlinearity terms down. In other words, at long times the nonlinearity contribution in Eq. (1) is negligible and the evolution becomes ballistic. Another thing we notice is how at a fixed time, *σ* (not the exponent) decreases with an increase in nonlinearity. This can be explained as follows: As *χ* increases, partial selftrapping of the excitation around the initial position increases as well and renormalizes the amount of radiation that can escape to infinity.

**Table 1:** Asymptotic exponent for the mean square displacement as a function of nonlinearity.

Nonlinearity Parameter	Propagation Exponent
0	2.00
0.5	2.01
1.0	1.99
1.5	1.98
1.0	1.99
1.5	1.98
2.0	2.14
2.5	2.09
3.0	2.12
3.5	2.21
4.0	2.33

## Conclusions

We have studied the nonlinear bulk and surface modes of the modified discrete nonlinear Schrödinger (mDNLS) equation. We have computed the linear stability of the lowest-order modes and have also computed the modulational stability of the uniform solution. We conclude that the fundamental bulk mode is stable with a stability curve extending all the way from the high nonlinearity region down to the linear band. In general, higher modes need a minimum nonlinearity to exist and can posses alternate stability as a function of power content. The surface modes, on the other hand, all need a minimum nonlinearity threshold to exist, with a stable fundamental mode. The existence of this threshold has been also observed in the DNLS case^30,31^. We have also estimated the dynamical barrier for the motion of a localized excitation across the lattice and obtained an approximate expression in terms of the amplitude, initial momentum and nonlinearity. The modulation stability of the special uniform solution was computed, concluding that the system is modulationally unstable. This means that the system favors the creation of nonlinear localized excitations (solitons). Finally, we computed the asymptotic transport exponent, by examining the mean square displacement of an initially localized excitation. We found that, at long times, and as a result of norm conservation, nonlinear effects becomes smaller and smaller and, as a result the propagation exponent becomes the ballistic one in the limit of an infinite time.

It should be mentioned that qualitatively similar results have been found previously for the complementary DNLS case. This is interesting and points out to the robustness of the phenomenology found here concerning the modes stability, the discrete soliton propagation, and the asymptotic propagation exponent. It also reinforces the idea of this type of discrete soliton as a robust entity capable of transporting an excitation across a generic discrete medium and thus, useful in several different physical systems.
